# Effects of nonoxynol-9 (N-9) on sperm functions: systematic review and meta-analysis

**DOI:** 10.1530/RAF-21-0024

**Published:** 2022-02-21

**Authors:** Murong Xu, Mingpeng Zhao, Raymond Hang Wun Li, Zhixiu Lin, Jacqueline Pui Wah Chung, Tin Chiu Li, Tin-Lap Lee, David Yiu Leung Chan

**Affiliations:** 1 Assisted Reproductive Technology Unit, Department of Obstetrics and Gynaecology, Faculty of Medicine, The Chinese University of Hong Kong, Hong Kong, China; 2 School of Biomedical Sciences, Faculty of Medicine, The Chinese University of Hong Kong, Hong Kong, China; 3 Department of Obstetrics and Gynaecology, The University of Hong Kong, Queen Mary Hospital, Hong Kong, China; 4 Hong Kong Institute of Integrative Medicine, Faculty of Medicine, The Chinese University of Hong Kong, Sha Tin, Hong Kong, China

**Keywords:** Nonoxynol-9, spermicide, progressive motility, vanguard sperm penetration distance, sperm function

## Abstract

**Objective:**

To summarize the currently available phase I and II clinical trials of the effects of nonoxynol-9 (N-9) on human sperm structure and functions.

**Methods:**

A systematic review and meta-analysis aiming to evaluate the spermicidal activity of N-9 on motility, was conducted in PubMed, EMBASE, and Cochrane databases by 10 March 2021. The counted numbers of progressive motile (PR) sperm in cervical mucus and the vanguard sperm penetration distances were analyzed. Other effects on sperm structures and physiological activities were reviewed as well.

**Results:**

In the pooled results, percentages or counted numbers of PR sperm decreased after the treatment of N-9. Vanguard sperm penetration distance was shortened in treated groups. N-9 has been confirmed to damage the structures of sperm, as well as other organelles like acrosome and mitochondria. The physiological activities such as generation of reactive oxygen species, superoxide dismutase activity, acrosin activity, and hemizona binding were all inhibited in the reviewed studies.

**Conclusions:**

N-9 has several impacts on sperm owing to its potency in reducing sperm motility and cervical mucus penetration, as well as other functional competencies.

**Lay summary:**

Nonoxynol-9 (N-9) has been used worldwide as a spermicide to kill sperm for more than 60 years but can cause side effects including vaginal irritation and can increase the rate of contraceptive failure. A detailed analysis of published literature aiming to evaluate the spermicidal activity of N-9 on sperm was carried out. In the pooled results, N-9 reduced the number of active sperm and the distance they traveled. It also caused damage to the structures of sperm and to the way the sperm acted and interacted with the egg. In conclusion, N-9 impacts on sperm in a number of ways that lead to sperm death and dysfunction.

## Introduction

Spermicide is a chemical barrier contraceptive used before intercourse for birth control, which has been used for thousands of years (Duffy & Archer 2018). The basis of spermicidal action is to immobilize or disrupt the plasma membrane of the sperm, so as to avoid sperm–egg interaction (Ingram *et al.* 2006). Nonoxynol-9 (N-9) is widely used as an active ingredient in various spermicides. Spermicides containing N-9 do not require a prescription but may be available online or in the drugstore from low to high prices without age restrictions. It may be available from some health centers as well (Iyer & Poddar 2008).

N-9 is a membrane disruptive agent that accounts for its contraceptive property (Rosenberg *et al.* 2008). It lyses the sperm membrane through the interaction with the membrane lipid, leading to sperm immobilization (Burke *et al.* 2010). N-9 is a product that is relied upon by 0.1% of contracepting women globally either as a primary method or in conjunction with barriers such as the diaphragms and cervical caps that help position it more effectively (Farley 2002, United Nations, Department of Economic and Social Affairs, Population Division 2015). The dosage forms can be in the foam, gel, films, and suppositories (Iyer & Poddar 2008) (Table 1). N-9 spermicides in the form of gel or foam can act immediately, while the dosage form of film or suppository should be inserted at least 15 min for dissolving. However, when using these products, new application should be applied for another intercourse, and some products also require an extra 6 h before douching although douching is never recommended. A bioadhesive delivery system has also been applied in some products to release the N-9 contents. After attaching to the vaginal epithelial surface, the gel starts releasing N-9 slowly at a stable pace, and the protection has been claimed to last for as long as 24 h (Sangi-Haghpeykar *et al.* 1996).

**Table 1:** Spermicide products containing N-9. Various deliver-9 products with different delivery systems and dosage options of N-9 are shown in the table, together with the directions for use.

Product	Form	Content	Required insert time	Instruction	Duration	Availability
VCF®	Film	28%	15 min	In contact with the cervix	Last for 3 h	Available
VCF®	Foam	12.5%	Less than 1 h; works immediately	Insert into vagina; wait at least 6 h for douching	–	Available
VCF®	Gel	4%	Works immediately	Insert into vagina	Up to 1 h	Available
Gynol II®	Gel	3%	Works immediately	Insert into vagina	Up to 1 h	Available
Conceptrol®	Gel	4%	Works immediately	Insert into vagina	Up to 1 h	Available
ContraSeed®	Suppository	100 mg	15 min	Insert and lie against the cervix	Up to 1 h	Available
Today®	Sponge	1000 mg	Works immediately	Wet sponge thoroughly and squeeze gently until sudsing; fold with dimple side inside; insert deeply into vagina with string loop on bottom end; wait 6 h before removing and avoid inserting for >30 h	24 h	Available
Encare®	Suppository	100 mg	At least 10 min	Insert into vagina; wait at least 6 h for douching	Up to 1 h	Not available
Advantage 24®	Gel	3.5%	Less than 30 min	Insert into vagina; release steadily in the bioadhesive delivery system attaching to the vaginal epithelial surface	24 h	Not available
Shur-Seal®	Gel	2%	At least 10 min; works immediately	Insert into vagina; wait at least 6 h for douching	–	Not available
Delfen®	Foam	12.5%	Up to 1 h	Insert into vagina; wait at least 6 h for douching	–	Not available

N-9 containing spermicide were convincingly found to be lethal to chlamydia and gonococcal organisms *in vitro* (Singh *et al.* 1972, Kelly *et al.* 1985, Patton *et al.* 1992). However, *in vivo*, studies have demonstrated no such protection (Roddy *et al.* 1998, 2002). Several clinical studies have raised concerns that N-9 containing spermicides might increase human immunodeficiency virus transmission and acquisition (Kreiss *et al.* 1992, Van Damme *et al.* 2002, Musekiwa *et al.* 2020). At the same time, the failure rate of it is higher when used as a contraceptive alone compared with many other contraceptive methods, with a Pearl index of over 20 per hundred women-year (Burke *et al.* 2010). These have prompted World Health Organization (WHO) to limit candidates for use of N-9 spermicides (World Health Organization 2010*b*).

While other products have been tested and one recently has been approved by the US Food and Drug Administration (FDA), N-9 is the most commonly used agent (Eisenberg 2020, Thomas *et al.* 2020). Its products constitute large proportions of the spermicides easily accessible in the market at affordable prices (Grimes *et al.* 2013). It can still be one of the options for women with a low risk of sexually transmitted diseases to prevent pregnancy. Phase I and II clinical trials studying the effects of N-9 focused on the sperm structure and functions. Sperm motility determines the ability of sperm to move toward the egg through the female reproductive tract. It is considered as the most important parameter to predict conception rate (Pasqualotto *et al.* 1999). Vanguard sperm penetration distance is also thought to have potential value in predicting IVF outcomes (De Geyter *et al.* 1988). Other sperm functions such as acrosome reaction and generation of reactive oxygen species (ROS), have also contributed to the pregnancy prediction (Ko *et al.* 2014, Tello-Mora *et al.* 2018). A holistic and up-to-date review is required to summarize the currently available phase I and II clinical trials to provide the knowledge foundation. We also want to find the inconstancies in the previous studies on N-9 and try to resolve the conflicts. Additionally, with the help of this review, we hope to provide new insights for further research on N-9. Thus, we conducted the systematic review and meta-analysis of published papers on functional mechanisms of N-9 on sperm structure and functions to update current understanding.

## Materials and methods

This study was conducted according to the guidelines of the Preferred Reporting Items for Systematic Reviews and Meta-Analyses and Cochrane Handbook for Systematic Reviews of Interventions (Version 6) (Moher *et al.* 2009, Higgins *et al.* 2019). This meta-analysis was registered at the International Prospective Register of Systematic Reviews (no. CRD42021227646).

### Search strategy

In order to conduct a comprehensive literature review, PubMed, EMBASE, and Cochrane Library databases were searched for eligible studies. All the relevant studies published before 10 March 2021 were included. The search strategies in the three databases were similar. We only used ‘Nonoxynol’ as the keyword or the medical subject headings (MeSH). The common synonyms of ‘Nonoxynol-9’ include ‘PEG-9 nonyl phenyl ether’ and ‘Tergitol NP-9’ (Wishart *et al.* 2018).

In PubMed, we used ‘Nonoxynol [MeSH Terms] OR PEG 9 nonyl phenyl ether OR PEG-9 nonyl phenyl ether OR Tergitol NP-9 OR Tergitol NP9’ as the search strategy. In EMBASE, we used ‘nonoxynol.mp. OR nonoxinol/ OR Tergitol NP9.mp. OR Tergitol NP-9.mp.’ as the search strategy. In Cochrane Library, ‘Nonoxynol’ was also used as the MeSH descriptors to explode all tress together with ‘PEG 9 nonyl phenyl ether’, ‘PEG-9 nonyl phenyl ether’, ‘Tergitol NP-9’, and ‘Tergitol NP9’. Additionally, the reference lists of the included studies were screened for more eligible studies.

### Eligibility criteria

We set inclusion criteria for considering studies in the systematic review and meta-analysis as listed: (i) Only mechanistic studies and no clinical efficacy trial data were included; (ii) *In vitro* studies were included where the sperm samples collected precoitally were all from healthy males aged over 18. Since the studies were conducted according to different versions of WHO guidelines, eligible studies only included participants with normal concentration and motility fulfilling the latest version. Eligible participants of *in vivo* studies were healthy, non-pregnant women (aged from 18 to 45 years) without known sensitivity to N-9 or the components contained in the products and had no risk of pregnancy. Before postcoital tests (PCTs), female participants were evaluated to be in the preovulatory period or had adequate cervical mucus. Sperm presence in the vaginal pool was considered as the false-negative control and presence in the cervical mucus was required; (iii) Spermicides containing N-9 were considered as the intervention, regardless of the forms and doses of N-9. The formulations could be in the forms of film, gel, foam, and suppository. Water, saline, phosphate buffer, DMSO, or Tyrode’s solution could be considered as the control group of *in vitro* studies. Personal baselines without any intervention were used as the control of the *in vivo* studies. Studies using N-9 together with other physical barriers were included to evaluate the synergistic effects as well; (iv) Sperm motility is one of the most important indicators to evaluate sperm quality, while only progressively motile (PR) sperm relates to the natural pregnancy. Sperm counts per high power field (HPF) or percentage of PR sperm in cervical mucus were considered as the primary outcome and vanguard sperm penetration distance was considered as the second outcome; (v) Other sperm function parameters such as acrosome reaction, hemizona binding, DNA integrity, and superoxide dismutase (SOD) activity were also included to evaluate the comprehensive functions; (vi) The longest time points were chosen if the studies were conducted at different time points.

We also set the exclusion criteria. Phase I and II clinical trials without control groups or using N-9 products as the control to confirm the efficacy of other chemicals were not included.

### Study selection and data extraction

All studies in the databases were reviewed by two investigators (Xu and Zhao) independently in accordance with the titles, abstracts, and full texts to evaluate the eligibility. All the articles satisfying the inclusion criteria were selected to conduct the systematic review and meta-analysis. Disagreements emerging in the process of the screening were resolved by all authors.

Available data were extracted from the screened articles meeting the selection criteria to conduct the meta-analysis. The information extracted from the studies included authors, publication time, study mode, quality of semen, sample size, the concentration of N-9, type of control, treatment duration, and reported sperm parameters. The primary outcome was the sperm counts/HPF or percentage of PR sperms in cervical mucus. The secondary outcome was the vanguard sperm penetration distance.

### Quality assessment

The biases of the articles were evaluated by the same two investigators (Xu and Zhao) independently using the ‘Risk Of Bias In Non-randomized Studies – of Interventions’ tool (ROBINS-I) (Sterne *et al.* 2016). The indicators included bias due to confounders, selection bias, bias in classification of intervention, bias due to deviations from intended interventions, bias due to missing data, bias in the measurement of outcomes, and bias in the selection of reported results. The disagreements in the process of the risk evaluation were also resolved by the third investigator (Chan).

### Statistical analysis

In order to analyze the effects of N-9 on sperm function, the meta-analysis was performed using the Review Manager 5.4 (Cochrane Collaboration, Oxford, UK) software with available data extracted from the screened articles. Mean differences (MDs) and 95% CIs were used to analyze the continuous data of the primary and secondary outcomes. These were also applied in subgroup analysis.

The heterogeneity among the studies was tested with Cochran's Q test (Higgins *et al.* 2003). The confidence level was set at *P*  = 0.1. I^2^ statistic was also performed to express and quantify the inconsistency of the results of studies. If the calculated I^2^ result met I^2^<50, showing that there was no statistically significant heterogeneity among the involved studies, the fixed model was used. Otherwise, the random-effects model was in use. A *P* -value of <0.05 was considered statistically significant. In some of the included studies, the s.d. was set as 0.000000001 since there were no variants among the groups (Mauck *et al.* 1997*a*, *b*, *c*, Schwartz *et al.* 2008, Mauck *et al.* 2017). The given s.e.m.was converted to s.d. by multiplying the square root of the sample size (Dunmire & Katz 1997*a*, *b*).

Additionally, Begg’s funnel plot was used to evaluate the publication bias of the studies. The symmetric shape indicated the precision of data and a small likelihood of publication bias.

## Results

### Meta-analysis of the effects of N-9 on sperm motility

#### Study selection

**Figure 1:**
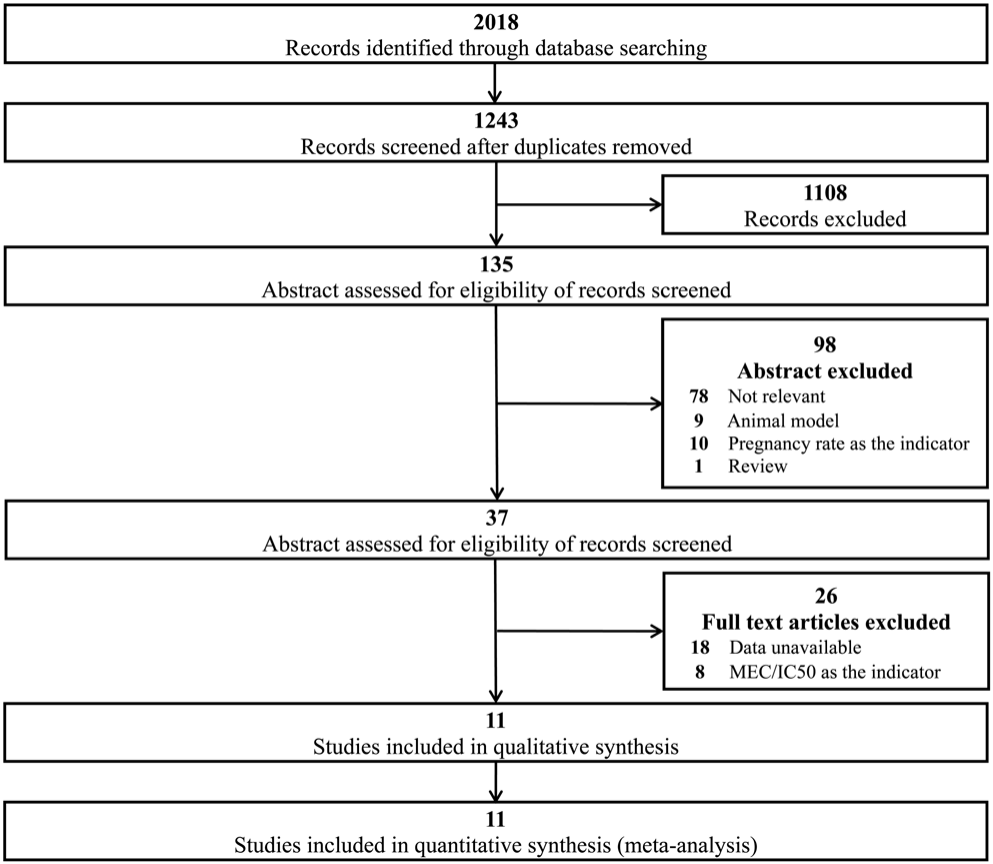
Flowchart of study selection for the systematic review.

There were 2018 relevant studies obtained in the three databases, including 886 in PubMed, 1031 in EMBASE, and 101 in the Cochrane database. After removing the duplications, 1243 studies were screened based on titles and abstracts, and 1108 studies were excluded. The remaining 135 studies were assessed further for eligibility based on the abstracts first. Among the 135 articles, 98 of them were removed due to different reasons (78 irrelevant articles, 9 animal studies, 10 using pregnancy rate as the indicator, and 1 review article). The full-text assessments excluded 8 which used MEC or LD50 as the indicators and 18 of them without available data. At last, we included 11 eligible articles in the systematic review and meta-analysis. The flowchart of the study selection was shown in Fig. 1.

#### Descriptions of studies

The 11 eligible papers included 7 *in vitro* tests with 96 participants and 11 PCTs with 229 participants. Seven experiments in four papers measured the vanguard sperm penetration distances after treatment with N-9. The remaining studies measured the PR sperm count/HPF in cervical mucus. The characteristics and detailed information about the reported sperm parameters in included studies were extracted and shown in Table 2.

**Table 2:** Characteristics of the included studies. We included 11 eligible articles and summarized the characteristics of the mode, semen specimen information, sample size, intervention concentration, control, and duration. Reported sperm parameters included progressive motility in cervical mucus and vanguard sperm penetration distance. Values are mean ± s.d. or as specified.

Reference	Mode	Sperm characteristics		Sample size		Duration (*h)*		Control	Intervention concentration	Reported sperm parameters (mm)	
		Concentration	Motility	Treated	Control	Treated	Control			Treated	Control
Vanguard sperm penetration distances											
Sharman *et al.* (1986)	*In vitro*	>40 × 10^6^/mL	>60%	3	3	2		Tyrode’s solution		%	%
									0.033 mg/mL	28.2 ± 4.1	27.3 ± 4.1
									0.33 mg/mL	13.4 ± 1.77	27.3 ± 4.1
									3.3 mg/mL	7.86 ± 2.3	27.3 ± 4.1
Dunmire & Katz (1997*b*)	*In vitro*	>40 × 10^6^/mL	>55%	4	4	>2	3	Water	100 mg/mL	41.5 ± 1.0	44.2 ± 0.3
Dunmire & Katz (1997*a*)	*In vitro*	>40 × 10^6^/mL	>55%	10	10	>2	3		100 mg/mL	%	%
								Water		40.4 ± 6	45.7 ± 4.1
								Saline		44.0 ± 2.8	49.3 ± 5.1
Mahony (2001)	*In vitro*	>100 × 10^6^/mL	>50%	5	5	4		Saline	5 mg/mL	2 ± 0.4	39 ± 6.7
PR sperm count/HPF											
Mauck *et al.* (1997*a*)	PCT	–		10	10	2.5 ± 0.4	2.5 ± 0.6	PB	VCF® (70 mg N-9)	0±0	22.2 ± 20.2
Mauck *et al.* (1997*c*)	PCT	–		10	10			PB		%	%
						2.8 ± 0.5	2.5 ± 0.5		VCF® (70 mg N-9)	0.5 ± 0.8	23.7 ± 26.7
						2.1 ± 0.5	2.5 ± 0.5		100 mg N-9	0.6 ± 0.9	23.7 ± 26.7
						2.5 ± 0.5	2.5 ± 0.5		130 mg N-9	0.9 ± 2.3	23.7 ± 26.7
Mauck *et al.* (1997*b*)	PCT	–		7	7			PB		%	%
						2.3 ± 0.4	2.5 ± 0.4		Femcap® with N-9	0.2 ± 0.4	18.0 ± 20.5
						2.6 ± 0.3	2.5 ± 0.4		Diagram with N-9	0 ± 0	18.0 ± 20.5
Ayotte & Colin (2002)	PCT	>20 × 10^6^/mL		5	9	<3 h		PB	Protectaid® sponge (0.125%N-9)	0.6 ± 1.1	17.8 ± 7.2
Amaral *et al.* (2004)	PCT	–		20	20	<2 h		PB	2% N-9	0.07 ± 0.23	17.94 ± 19.91
Schwartz *et al.* (2008)	PCT					2–3 h		PB		%	%
		–		13	14				SILCS (metal)+N-9	0 ± 0	12.5 ± 8.8
		–		8	14				SILCS (polymer spring)+N-9	0 ± 0	12.5 ± 8.8
Mauck *et al.* (2017)	PCT	–		9	9	2–3 h		PB	Caya®+3% N-9	0.6 ± 1.1	22.5 ± 33.4

PB, personal baseline; PR, progressively motile; PCT, postcoital tests.

We measured seven types of potential biases in each paper. The judgment was explained by ‘low’, ‘moderate’, and ‘high’ risk. The risk of biases due to confounders were low in all studies. As to the bias in the selection of participants into the studies, all studies fulfilled strict criteria to select participants. Risks of biases due to the classification of intervention and selection of the reported results were both low in all studies. One study indicated unwanted outcomes in some participants, which might contribute to the bias due to deviation from intended interventions (Ayotte & Colin 2002). Biases due to missing data might be high in one study where some participants did not complete the tests (Schwartz *et al.* 2008). The risk of bias in the measurement of outcomes was low in all studies. Thus, the overall biases were low in all studies. The quality of the included studies was evaluated with the ROBINS-I tool in Table 3.

**Table 3:** Risk of bias assessment using ROBINS-I. The quality of the included papers was evaluated by the ROBINS-I tool using the judgment of ‘low’, ‘moderate’, and ‘high’ risk. The overall biases of all the papers included were considered at low risks.

	Sharman *et al*. (1986)	Dunmire & Katz (1997*b*)	Dunmire & Katz (1997*a*)	Mahony (2001)	Mauck *et al.* (1997*a*)	Mauck *et al*. (1997*c*)	Mauck *et al.* (1997*b*)	Ayotte & Colin (2002)	Amaral *et al.* (2004)	Schwartz *et al*. (2008)	Mauck *et al*. (2017)
Bias due to confounding	L	L	L	L	L	L	L	L	L	L	L
Bias in selection of participants into the study	L	L	L	L	L	L	L	L	L	L	L
Bias in classification of interventions	L	L	L	L	L	L	L	L	L	L	L
Bias due to deviations from intended interventions	L	L	L	L	L	L	L	H	L	L	L
Bias due to missing data	L	L	L	L	L	L	L	L	L	H	L
Bias in measurement of outcomes	L	L	L	L	L	L	L	L	L	L	L
Bias in selection of the reported result	L	L	L	L	L	L	L	L	L	L	L
Overall	L	L	L	L	L	L	L	L	L	L	L

L, low risk; M, moderate risk; H, high risk.

#### Primary outcome – progressive motility in cervical mucus

**Figure 2:**
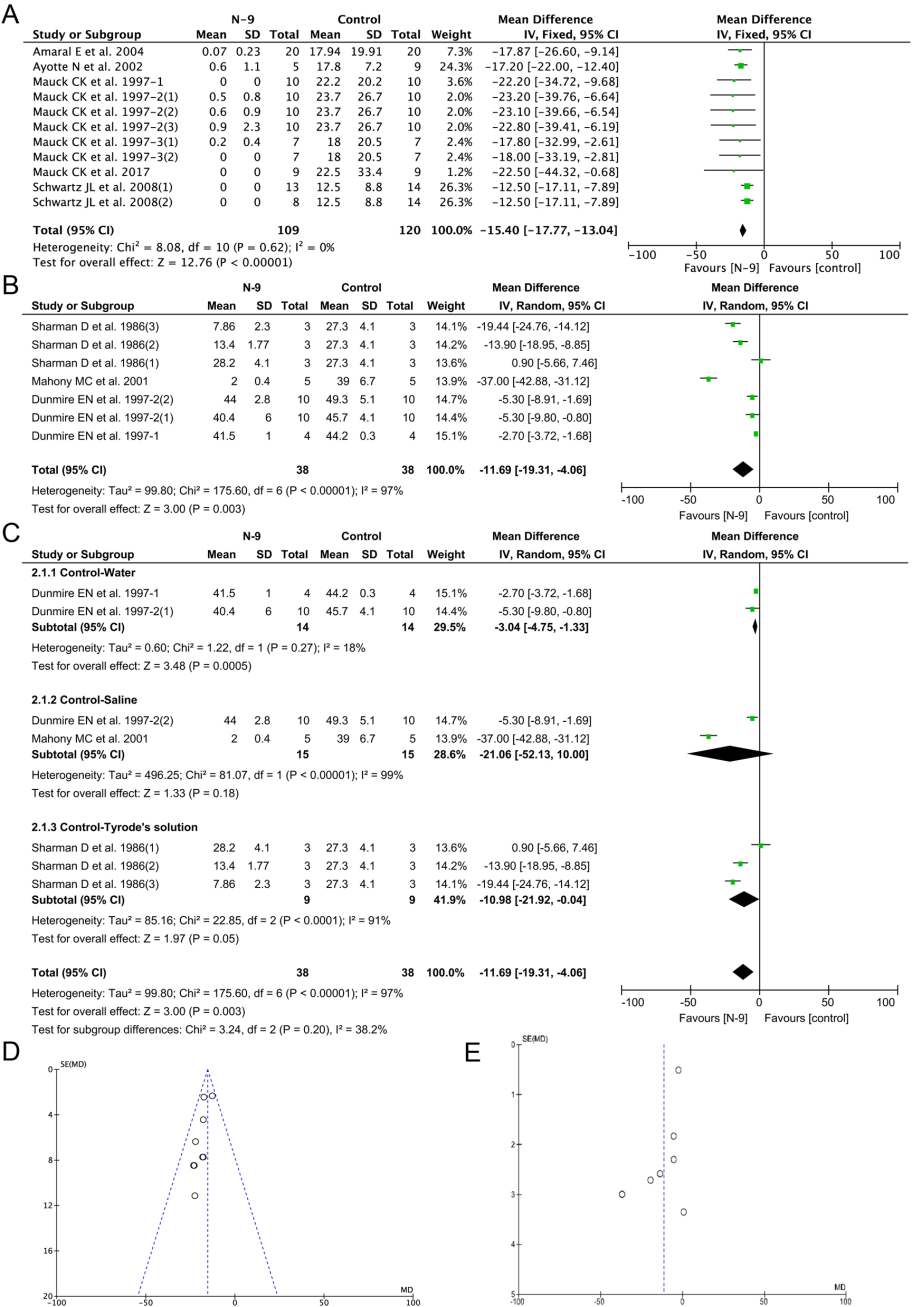
Meta-analysis forest plots and funnel plots.

Sperm motility determines the ability of sperm to move toward the egg through the female reproductive tract. It is considered the most important parameter to predict conception rate (Pasqualotto *et al.* 1999). Sperm is classified as PR, non-progressively motile (NP), and immotile (World Health Organization 2010*a*). Therefore, progressive motility in the cervical mucus was chosen as the primary outcome. Eleven experiments in eight studies evaluated the PR sperm count/HPF or percentage in cervical mucus (Fig. 2A). The overall heterogeneity of these studies was large (I^2^= 0%), so the fixed effects model was applied. The pooled MD and 95% CI were −15.40 and −17.77 to −13.04 indicating a significant decrease in PR sperm after treatment with N-9. With N-9 intervention, the mean of sperm counts in the treated group was at least 15 lower than the mean of sperm counts in the control group. The results of the meta-analysis provided evidence that N-9 was effective in decreasing sperm motility in the cervical mucus.

#### Secondary outcome – vanguard sperm penetration distance

The *in vitro* sperm–mucus penetration test evaluates the sperm quality by measuring the distance that sperm swim up in the cervical mucus (De Geyter *et al.* 1988). The vanguard sperm penetration distance is shown to have positive associations with fertility (Murase *et al.* 1990). The vanguard sperm penetration distance used in our analysis is the distance between the foremost sperm and the mucus interface at the departure point of the treatment in the capillary tube. Seven experiments from four papers evaluated the vanguard sperm penetration distances, including 76 participants (Fig. 2B). In the pooled analysis of the vanguard sperm penetration distances, the mean difference was −11.69 with 95% CI −19.31 to −4.06. Since the heterogeneity was high with I^2^= 97%, a random-effects model was used. Sperm treated with N-9 had significantly shorter penetration distances compared with those in the control group (*P*  = 0.003).

The stratified meta-analysis of vanguard sperm penetration distance was based on the different controls (Fig. 2C). Among the experiments, two used water as control, and another two used saline as control. The remaining three experiments used Tyrode’s solution as control. The pooled MDs (95% CIs) of the three different controls were −3.04 (−4.75 to −1.33), −21.06 (−52.13 to 10.00), and −10.98 (−21.92 to −0.04) respectively. There were significant differences in the experiments using water and Tyrode’s solution as the controls, but no difference in the experiments using saline as the control. Among the experiments with water and Tyrode’s solution as control, the penetration distances in the N-9-treated group were shorter than those in the control groups.

In the stratified meta-analyses, N-9 significantly reduced vanguard sperm penetration distance only when water or Tyrode’s solution, but not saline as the medium. Among the three media used in controls, Tyrode’s solution should be the most suitable as it provides isosmotic loading and the nutrition needed by sperm.

#### Publication bias

The publication biases were shown in funnel plots (Fig. 2D and E). Deviation from the funnel plots implicated the significant presence of the publication biases. The symmetric funnel-shaped distribution of the progressive motility in cervical mucus demonstrated that the data sets behaved well with less likelihood of publication bias, while the non-symmetric funnel-shaped distribution of vanguard sperm penetration distances indicated more likelihood of publication bias.

### Effect of N-9 on other mechanisms of sperm function

A summary of the studies on the mechanisms of N-9 on human sperm function which cannot be analyzed quantifiably is shown in Table 4.

**Table 4:** Summary of the functional mechanisms of N-9 on sperm. The table showed the studies focusing on the effects of N-9 on the viability, motility characteristics, acrosome intactness, acrosin activity, sperm membrane, other organelles, and other aspects of human sperm.

Reference	Sperm		Sample size	N-9 concentration	Descriptions of the effect of N-9
	Concentration	Motility			
Viability					
Jain *et al.* (2010)	>65 × 10^6^/mL	>70%	–	500 μg/mL	Necrosis in nearly 90% of sperm; viable sperm cell populations fell to ~2%.
Shah *et al.* (2005)	–	–	10	–	Near 0% viability at 0.08 mg/mL.
Ping *et al.* (2005)	>15 × 10^6^/mL	–	15	0.5, 0.25, 0.125, and 0.0625 mg/mL	0% viability within 30 min at ≥0.25 mg/mL; 0% viability within 20 s at 0.5mg/mL
Jain *et al.* (2009)	>60 × 10^6^/mL	>75%	–	–	Necrosis induction in a significant number of sperm nonspecifically.
Motility characteristics					
Centola (1998)	–		6	2000, 1000, 100, 20, 10 μg/mL	0% motility at 2000–20 μg/mL, 6.5 ± 5.74% motility at 10 μg/mL; 0 progressive velocity at all doses except at 10 μg/mL (8.2 ± 5.5), with a significant decrease compared to fresh (46.7 ± 4.03); significant decrease of hyperactivation by N-9 (0–0.3 ± 0.3%).
Dunmire & Katz (1994)	>60 × 10^6^/mL	>50%	6	0, 15, 30, 45, and 60 μg/mL	Significant decreases in motility, LIN, and VSL (*P* < 0.005); significant increase in MAD (*P* < 0.001); slight decrease in VCL (*P* < 0.1).
Dunmire & Katz (1997*a*)	>40 × 10^6^/mL	>55%	9	–	Significant decrease in MOT (*P* < 10−5); significant decrease in ALH (*P* < 0.01) for N9-saline treatment; significant decreases in LIN, VCL, VSL (*P* < 0.01), and significant increase in MAD (*P* < 0.05) for N9-saline andwater treatment.
Mahony (2001)	>100 × 10^6^/mL	>50%	5	5 mg/mL	Very significant effect on all sperm motility characteristics, VCL, ALH, and LIN (*P*< 0.01).
Zaïri *et al.* (2013)	–		20	5–200 μg/mL	0% motility at ≥100 μg/mL.
Zairi *et al.* (2008)	–		50	0–500 μg/mL	0% motility at ≥100 μg/mL.
White *et al.* (1995)	>20 × 10^6^/mL	>40%	>50	5, 50, and 500 μg/mL	No significant effect on human sperm motility at 5 μg/mL; significant (P < 0.01) reduction of motility at 50 μg/mL; complete stop in all sperm movement within 1 min at 500 μg/mL.
Srivastava & Coutinho (2010)	–		8	–	Significant effect on sperm motility at a dose of 50 µM.
Louis & Pearson (1985)	–		6	–	Decreased motility with the increasing concentration.
Lee *et al.* (1996)	>20 × 10^6^/mL	>50%	–	0.050, 075, 0.1, 0.125, 0.15, and 0.175 mg/mL	Decreased motility with the increasing concentration.
Harrison & Chantler (1998)	>15 × 10^6^/mL	>40%	5	0.2, 0.4, and 0.6 mg/mL	0% motility at ≥0.4 mg/mL.
Shah *et al.* (2005)	–		10	–	Decreased motility with the increasing concentration.
Ping *et al.* (2005)	>15 × 10^6^/mL		15	0.5, 0.25, 0.125, and 0.0625 mg/mL	0% motility at ≥0.25 mg/mL within 20 s; 0% motility at ≥0.125 mg/mL in 30 min.
Acrosome intactness and acrosin activity					
Centola (1998)	–		6	2000, 1000, 100, 20, and 10 μg/mL	Complete break down and release of the acrosomal contents at all doses (16 ± 2.6–26 ± 6.0% RITC+; 73.3 ± 4.8–83.7 ± 2.6% RITC−).
Harrison & Chantler (1998)	>15 × 10^6^/mL	>40%	44	0.2, 0.4, and 0.6 mg/mL	Loss of the acrosomal structure.
Xia *et al.* (2020)	–			40 mg/mL	No intact acrosome from a weak fluorescence strap that appeared in the equatorial zone.
Wilborn *et al.* (1983)	–		30	0.05, 0.1, 0.25, 0.5, 1.0, 2.5, 5.0, and 10.0%	Damage in acrosomal membrane complex, which ranged from vesiculations to complete obliteration; not always affected post-acrosomal membrane.
Schill & Wolff (1981)	–		–	0.05%	Missed acrosomal membrane complex including equatorial segment.
Müller-Esterl & Schill (1982)	–		–	0.05–1%	Gelatin film method (acrosin activity): complete prevention gelatin lysis at 0.05–1% N-9; moderate halo formation at 0.001–0.01%; completely missed halo formation at 0.05–1%.
Sperm membrane and other organelles					
Harrison & Chantler (1998)	>15 × 10^6^/mL	>40%	44	0.2, 0.4, and 0.6 mg/mL	Severely damaged membrane organization; partial dissolution of plasma membrane; partly exposed nucleus.
Jain *et al.* (2010)	>65 × 10^6^/mL	>70%	–	500 μg/mL	Significant (*P*< 0.001) depolarization of plasma membrane potential.
Xia *et al.* (2020)	–		–	40 mg/mL	Rare appearance of spermatozoa with a swollen tail; deconstruction of membrane permeability; leakage of cytoplasm.
Wilborn *et al.* (1983)	–		30	0.05, 0.1, 0.25, 0.5, 1.0, 2.5, 5.0, and 10.0%	Destruction of the cell membrane of the neck; absence of midpiece membrane; extirpated mitochondria of the midpiece and exposed fibers in approximately 25% of sperm; vesiculations as the first evidence of all membrane damage; loose and detached membranes then; membrane of the tail not always affected.
Thompson *et al.* (1996)	–		–	0.01, 0.02, 0.03, and 0.04%	Increase of sperm cell permeabilization.
Shah *et al.* (2005)	–		10	–	Membrane perturbation and disruption.
Schill & Wolff (1981)	–		–	0.05%	Completely removed plasma membrane from head to the end piece; appearance of swollen, break, discontinuous nuclear membrane; enlarged space between the nucleus and the nuclear membrane; swollen chromatin structures in some sperm and nuclear decondensation in others; missed cytoplasm in the neck region and the middle piece; appearance of monolayer membrane of the mitochondria instead of bilaminar membrane; empty interior of the mitochondria or containing a fine granular material and disappearance of normal cristae.
Lakshmi *et al.* (2008)	>60 × 10^6^/mL	>75%	–	0.05%	Physiological damage of sperm membrane.
Others					
Jain *et al.* (2010)	>65 × 10^6^/mL	>70%	–	500 μg/mL	*Intracellular pH of human sperm:* Significant (*P*< 0.01) intracellular acidification of human sperm at 20 μg/mL;*ROS generation:* Significant snduction of ROS (*P*< 0.001) in human sperm;*SOD activity:* significant inhibition of SOD activity of human sperm (*P*< 0.01);*Sperm dyenin ATPase activity:* no significant changes in dyenin ATPase activity which provides motor energy to sperm;*Tyrosine phosphorylation:* visibly significant inhibition of tyrosine phosphorylation in human sperm;*Hemizona assay:* a potent inhibition of sperm-zona pellucida binding: the index was 10.

Linearity-LIN, Straight line velocity-VSL, Mean angular displacement-MAD, Curvilinear velocity-VCL, Motility- MOT, Amplitude of lateral head displacement-ALH, Rhodamine isothio- cyanate conjugated-RITC, Reactive Oxygen Species-ROS, Superoxide dismutase-SOD.

#### Effects on viability and motility characteristics

Studies showed that N-9 treatment resulted in significantly reduced sperm viability to nearly 0% even at a low concentration (Ping *et al.* 2005, Shah *et al.* 2005, Jain *et al.* 2009, 2010), mainly by inducing necrosis in the majority of sperm (Jain *et al.* 2010).

As to the effects on motility characteristics, many studies showed the ability of N-9 to interfere with sperm motility even at a low concentration (Louis & Pearson 1985, Dunmire & Katz 1994, 1997*a*, White *et al.* 1995, Lee *et al.* 1996, Centola 1998, Harrison & Chantler 1998, Mahony 2001, Ping *et al.* 2005, Shah *et al.* 2005, Zairi *et al.* 2008, Srivastava & Coutinho 2010, Zaïri *et al.* 2013). Three of them also evaluated motility parameters by computer-assisted sperm analysis and showed that N-9 treatment significantly reduced straight- line velocity (VSL), curvilinear velocity (VCL), linearity (LIN), and amplitude of lateral head displacement (ALH), and increased mean angular displacement (MAD) (Dunmire & Katz 1994, 1997*a*, Mahony 2001) (Supplementary data 1, see section on supplementary materials given at the end of this article). VSL, VCL, LIN, and ALH have been reported to positively associate with fertility, while MAD shows a negative correlation (King *et al.* 2000, Youn *et al.* 2011). For example, The increase of ALH after semen preparation is beneficial to the pregnancy outcome (Fréour *et al.* 2010). Additionally, the level of hyperactivation was inhibited significantly as well after treatment with N-9 (Centola 1998).

#### Effects on sperm acrosome

Five studies provided information on acrosome intactness, together with one study focusing on acrosin activity. Studies evaluating the acrosome structure all gave the conclusions that N-9 damaged the acrosomal membrane complex, resulting in the release of the acrosome enzymes, and the sperm structure was even found to break down completely since 10μg/ml (Schill & Wolff 1981, Wilborn *et al.* 1983, Centola 1998, Harrison & Chantler 1998, Xia *et al.* 2020). Although the release of the acrosomal enzymes contributes to penetrating the zona pellucida, it has also been found that the activity of the enzymes was inhibited at the same time. The gelatin film method was used to evaluate the acrosin activity after the N-9 treatment. The complete prevention of gelation lysis after N-9 treatment indicated the influence on acrosin activity, even at the lowest concentration of 0.05% (Müller-Esterl & Schill 1982). Among the studies, the missed acrosomal structure was also found in the equatorial zone, and the damage was presented in the form of vesiculations to complete obliteration (Schill & Wolff 1981, Xia *et al.* 2020). After an acrosome reaction, the forepart of the sperm is only covered by the inner acrosome membrane. For the further binding with the oolemma, the post-acrosomal membrane plays a critical role (Singh 2014). However, the post-acrosomal membrane was not always affected by the N-9 treatment (Wilborn *et al.* 1983).

#### Effects on sperm membrane integrity

Effects of N-9 on sperm membrane were reported. Severe necrosis was observed, and it began with membrane disruption (Shah *et al.* 2005, Jain *et al.* 2010). Severe damage of cell membrane organization was observed (Schill & Wolff 1981, Wilborn *et al.* 1983, Harrison & Chantler 1998, Shah *et al.* 2005, Lakshmi *et al.* 2008). Vesiculations would appear first during the damage caused by N-9, and then the membranes would start loosening and detaching (Wilborn *et al.* 1983). However, the tail membrane was not always influenced by N-9, and spermatozoa with a swollen tail were rarely seen (Xia *et al.* 2020). At the same time, the membrane permeability was deconstructed, and it would be increased by N-9 treatment, together with the significant depolarization of plasma membrane potential (Thompson *et al.* 1996, Jain *et al.* 2010, Xia *et al.* 2020). After membrane disruption, leakage of cytoplasm and further disruption of the nuclear membrane with enlargement of the nuclear space occurred (Schill & Wolff 1981, Xia *et al.* 2020). Then, the nucleus was partly exposed and swollen chromatin structures or nuclear decondensation could be seen (Schill & Wolff 1981, Harrison & Chantler 1998). Additionally, two studies reported the effects on mitochondria. N-9 treatment turned the bilaminar membrane of mitochondria into monolayer, and the interior became empty with the production of granular materials and the disappearance of normal cristae (Schill & Wolff 1981). Mitochondria might also be extirpated by N-9 directly (Wilborn *et al.* 1983).

#### Other aspects

The effects of N-9 on other aspects such as generation of ROS, the activity of SOD, and sperm dynein ATPase were reviewed in the study by Jain *et al*. as well. When treated with N-9, a significant inhibition could be seen in the tyrosine phosphorylation, which is associated with sperm capacitation. The significant suppression could also be seen in the activity of SOD, which plays an important role in cell metabolism. As to the ROS generation, the result showed that it was induced significantly, which might lead to oxidate stress which damages cell structure. When treated by N-9 at 500 μg/mL, sperm–zona pellucida binding was inhibited potently. Additionally, significant intracellular acidification of human sperm could be seen at a low concentration of N-9 and acidic pH is associated with decreased motility and capacitation (Zhou *et al.* 2015). However, the activity of sperm dynein ATPase, which provides energy support to sperm did not exhibit significant changes (Jain *et al.* 2010).

## Discussion

Nonoxynol-9 has been used as a spermicide worldwide for more than 60 years. In order to better understand the mechanisms of N-9 on various aspects of sperm functions, a holistic and up-to-date review was conducted.

In this systematic review and meta-analysis, we included 11 eligible studies to assess the impacts of N-9 on sperm with the changes in numbers of PR sperm in cervical mucus demonstrated by PCT in *in vivo* studies as the primary outcome and vanguard sperm penetration distances in *in vitro* studies as the secondary outcome. The primary result showed that, in cervical mucus, the number of PR sperm per HPF decreased significantly after N-9 treatment when compared with the personal baselines. As one meta-analysis indicates that vanguard sperm penetration distance has low accuracy in evaluating sperm motility compared with sperm count/HPF, this was taken as the secondary outcome (Ola *et al.* 2003). The vanguard sperm penetration distance in *in vitro* tests was shorter after the N-9 treatment when compared with the control.

The effects of N-9 on other functional mechanisms of the human sperm were also reviewed. Briefly, N-9 disrupted the plasma membrane and resulted in the increase of cell permeabilization and leakage of the cell contents, followed by damage of the nuclear membrane (Schill & Wolff 1981, Thompson *et al.* 1996, Harrison & Chantler 1998, Xia *et al.* 2020). The nucleus exposed to N-9 would experience swollen chromatin structures or nuclear decondensation (Schill & Wolff 1981). During this process, N-9 also disrupts the organelles of the sperm-like acrosome in the sperm head and mitochondria in the midpiece (Schill & Wolff 1981, Wilborn *et al.* 1983, Centola 1998, Harrison & Chantler 1998, Xia *et al.* 2020). Inhibition of acrosin also impacts penetration through the zona pellucida (Müller-Esterl & Schill 1982). Additionally, some other physiological activities like ROS generation, SOD activity, tyrosine phosphorylation, and hemizona binding were all altered (Jain *et al.* 2010). With the disruption to the structures and physiological activities, the viability and motility were affected in different facets, contributing additional functional mechanisms on top of the effect on sperm motility.

This meta-analysis has several limitations: (i) The number of the studies and the sample sizes were limited, and some studies only contained several participants; (ii) In our meta-analysis, only the sperm motility and vanguard sperm penetration distances were included. Other parameters such as viability, kinematic characteristics, and physiological activities were only described qualitatively without numerical data; (iii) Different studies made use of N-9 in different forms and in different concentrations. It has been shown that the efficacy of N-9 has associations with the dose but on the other hand, a high dose may contribute to a higher risk of vaginal irritation (Raymond *et al.* 2004). We did not do stratified analysis based on the concentration of the treatment due to the high degree of variations; (iv) In all *in vitro* tests, seven experiments in four papers used three different controls, which might increase the heterogeneity among the experiments. Also, the anisosmotic environment when using water as the control might cause variations. 5) The studies included varied from 1986 to 2017 and they used different WHO guidelines. We included studies whose given parameters fulfilled the latest version, while the given parameters were not integrated. It is worth noting that although only one study used the latest edition the rest followed the earlier edition of WHO semen analysis manual so might result in coherent parameters.

Up to date, scientists have put forward many alternatives to replace N-9 in spermicides. For example, tideglusib, a protein from Ricinus communis, desgalactotigonin, and bivittoside D from *Bohadschia vitiensis* have been considered as potential spermicides in animal models (Nithya *et al.* 2012, Chakraborty *et al.* 2014, Chen *et al.* 2019). Combinations of N-9 and propranolol or polyvinylpyrrolidone both showed a complementary effect to achieve a complete cessation of sperm motility (White *et al.* 1995, Fowler *et al.* 2003). In 2020, FDA approved a new contraceptive vaginal pH modifier without N-9, which kills the sperm by changing the vagina acidity. It is also confirmed to have higher efficacy compared with N-9 products, and even lower the risk of gonorrhea and chlamydia infections (Chappell *et al.* 2021). The latest review on N-9 has been published for more than ten years and none of the reviews emphasized on functional mechanisms of N-9 on human sperm. A comprehensive understanding of the N-9 on sperm structures and functions can benefit the further contraceptive targeting.

Since it has been confirmed to be effective in killing sperm, more research can be done to explore complementary agents like anti-microbiotics or other agents which can change the vagina environment to achieve the goal of preventing pregnancy more effectively and to confer non-contraceptive benefits such as microbicidal effects. In conclusion, N-9 has several effects on sperm owing to its potency in reducing sperm motility and cervical mucus penetration, as well as other functional competencies.

## Supplementary Material

Supplementary Figure 1

## Declaration of interest

Raymond Hang Wun Li is an Associate Editor of Reproduction and Fertility. Raymond Hang Wun Li was not involved in the review or editorial process for this paper, on which he is listed as an author. The other authors declare no conflict of interest.

## Funding

This work was supported by Right Pearl Limited, research contract TR1914524.

## Author contribution statement

M X and M Z reviewed the studies and analyzed the data. M X drafted the manuscript, and R H W L and D Y L C edited it. Z L, J P W C, T C L, and D Y L C supervised the project.
