# Supplementary material for: Effects of nonoxynol-9 (N-9) on sperm functions: systematic review and meta-analysis
Source: Reprod Fertil. 2022 Feb 21;3(1):R19–33. doi: 10.1530/RAF-21-0024 (PMC8956826; doi:10.1530/RAF-21-0024)
Supplement: Supplementary Figure 1 [file supplementary_figure_1.pdf]

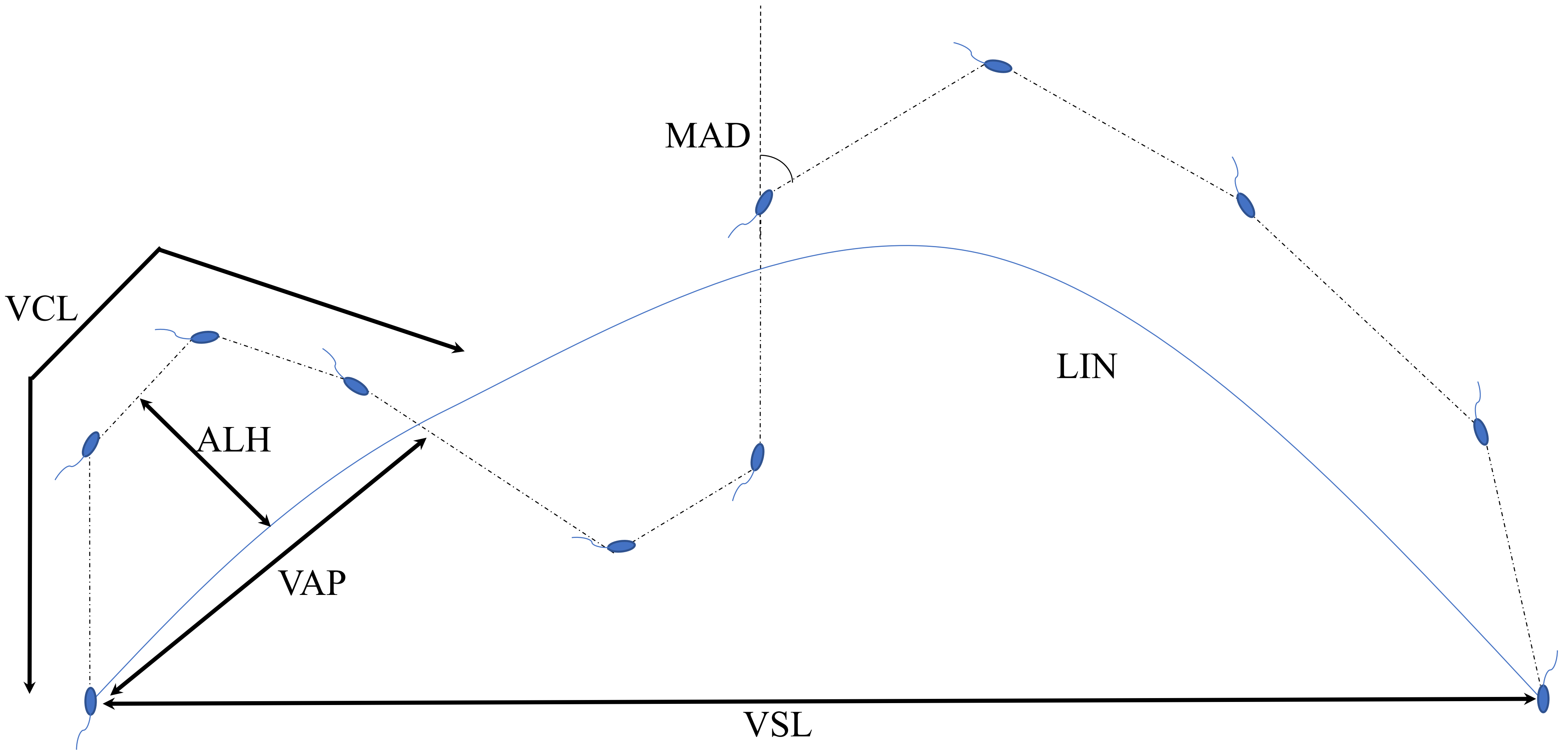

VSL: straight line velocity
VCL: curvilinear velocity
LIN: linearity of the curvilinear trajectory (VSL/VCL × 100)
ALH: amplitude of lateral head displacement
MAD: mean angular displacement
